# Haemolysis and Perturbations in the Systemic Iron Metabolism of Suckling, Copper-Deficient *Mosaic* Mutant Mice – An Animal Model of Menkes Disease

**DOI:** 10.1371/journal.pone.0107641

**Published:** 2014-09-23

**Authors:** Małgorzata Lenartowicz, Rafał R. Starzyński, Wojciech Krzeptowski, Paweł Grzmil, Aleksandra Bednarz, Mateusz Ogórek, Olga Pierzchała, Robert Staroń, Anna Gajowiak, Paweł Lipiński

**Affiliations:** 1 Department of Genetics and Evolution, Institute of Zoology, Jagiellonian University, Kraków, Poland; 2 Department of Molecular Biology, Institute of Genetics and Animal Breeding, Polish Academy of Sciences, Magdalenka, Poland; 3 Department of Cell Biology and Imaging, Institute of Zoology, Jagiellonian University, Kraków, Poland; University of Florida, United States of America

## Abstract

The biological interaction between copper and iron is best exemplified by the decreased activity of multicopper ferroxidases under conditions of copper deficiency that limits the availability of iron for erythropoiesis. However, little is known about how copper deficiency affects iron homeostasis through alteration of the activity of other copper-containing proteins, not directly connected with iron metabolism, such as superoxide dismutase 1 (SOD1). This antioxidant enzyme scavenges the superoxide anion, a reactive oxygen species contributing to the toxicity of iron *via* the Fenton reaction. Here, we analyzed changes in the systemic iron metabolism using an animal model of Menkes disease: copper-deficient *mosaic* mutant mice with dysfunction of the ATP7A copper transporter. We found that the erythrocytes of these mutants are copper-deficient, display decreased SOD1 activity/expression and have cell membrane abnormalities. In consequence, the *mosaic* mice show evidence of haemolysis accompanied by haptoglobin-dependent elimination of haemoglobin (Hb) from the circulation, as well as the induction of haem oxygenase 1 (HO1) in the liver and kidney. Moreover, the hepcidin-ferroportin regulatory axis is strongly affected in *mosaic* mice. These findings indicate that haemolysis is an additional pathogenic factor in a mouse model of Menkes diseases and provides evidence of a new indirect connection between copper deficiency and iron metabolism.

## Introduction

Copper and iron are essential biometals, both existing in two main oxidation states, i.e. Cu(I)/Cu(II) and Fe(II)/Fe(III). The extensive range of redox potential available to both metals by varying their interactions with coordinating ligands, as well as their capacity to participate in one-electron transfer reactions, are the reasons why copper and iron are essential for almost all living organisms. Both metals serve as cofactors for enzymes that catalyze diverse redox reactions underlying fundamental metabolic processes, including respiratory oxidation, DNA, microRNA and neurotransmitter synthesis, oxygen radical scavenging and connective tissue formation [1], [2].

In mammals, iron is much more abundant than copper [3], mainly because it is an essential component of haem in the oxygen-carrying molecules haemoglobin and myoglobin. On the other hand, the biological activities of copper and iron also have a toxic aspect, since both cuprous [Cu(I)] and ferrous [Fe(II)] ions are able to react with hydrogen peroxide in the Fenton cycle, yielding the highly destructive hydroxyl radical, the most reactive free radical species known. It is therefore vital that copper and iron are carefully sequestered and transported to their intracellular destinations. Regulatory systems have evolved to maintain iron [1] and copper [2] homeostasis. Interestingly, some pathways of iron homeostasis largely depend on the activity of copper-containing ferroxidases such as caeruloplasmin (Cp) and hephaestin. The former is mainly synthesized in hepatocytes in the form of apo-Cp. Incorporation of copper into apo-Cp results in the formation of the redox-active holoenzyme and is mediated by the P-type ATPase, ATP7B during transit through the trans-Golgi network. Cp is then secreted to the circulation where it functions to deliver copper to other tissues. Although Cp-bound copper accounts for about 90% of the total serum copper, it is not indispensable for efficient copper distribution in the body [3], [4], [5]. It has been shown that an alternative spliced glycosylphosphatidylinositol (GPI) variant of Cp (Cp-GPI) originally characterized in the brain [6] is of particular importance for iron metabolism [3], [5]. Cp-GPI cooperates with ferroportin (Fpn), the sole cellular iron exporter known in mammals [7], to facilitate the movement of iron out of cells. First, ferrous ions transported into the circulation by ferroportin are oxidized by Cp-GP1 and ferric ions are then bound by transferrin. Second, Cp-GPI is required for the stability of cell surface ferroportin [8].

Copper deficiency has not been reported to significantly and directly influence ferroportin gene expression [9], [10], but on the other hand, treatment of J774 macrophage cells with copper has been shown to induce ferroportin and to increase iron efflux from these cells [11]. The cooperation Cp-GPI and ferroportin is crucial for iron recycling by reticuloendothelial cells and iron release by hepatocytes [3]. The role of Cp in iron metabolism has been reported in the congenital human disease acaeruloplasminemia, in which mutations in the caeruloplasmin gene lead to hypoferraemia and iron loading in the liver [12], [13]. Hepatic iron overload also occurs in acquired copper deficiency and is likely mediated by hypocaeruloplasminemia [14]. Furthermore, iron overload was reported in Wilson disease, a genetic disorder caused by the mutation in *ATP7B* gene. The lack of ATP7B activity in patients with Wilson disease results in impaired copper loading to apo-Cp and in consequence in decreased Cp ferroxidase activity [15].

Hephaestin shares 50% homology with Cp and is a ferroxidase required for the egress of iron from intestinal enterocytes into the circulation. Sex-linked anaemia (sla) mice, carrying mutations in the gene encoding hephaeastin develop moderate to severe microcytic hypochromic anaemia [16]. Systemic iron deficiency has been also reported in copper-deficient mice displaying decreased hephaestin ferroxidase activity in the intestine [17]. Evidence of the nutritional requirement for copper in maintaining iron homeostasis dates back to the study of Hart et al. in 1928, in which copper was found to be necessary for haemoglobin formation in rats fed on a copper-deficient milk-based diet [18]. Numerous examples of the correlation between copper deficiency and poor iron status have been provided by studies on rapidly growing suckling piglets [19], [20].

In mammals, dietary copper is transferred across the apical membrane into enterocytes in its reduced (cuprous) form by copper transporter 1. Transport through the enterocyte basolateral membrane is mediated by ATP7A, a P-type ATPase copper transporter. In the blood copper is bound to albumin or α2-macroglobulin and is delivered to the liver *via* the portal circulation [21], [22]. Copper deficiency is associated with Menkes disease, a life-threatening pathology in man, which is due to mutations in the *ATP7A* gene, resulting in defective functioning of ATP7A [21], [23]. The general pattern of copper content in the organs of patients with Menkes disease indicates that copper is scarce in the brain, liver and heart, while it accumulates in the small intestine and kidneys [23]. This is consistent with the role of ATP7A as a membrane protein transporting copper out of duodenal enterocytes [21]. In other cells, under physiological conditions, this protein is primarily localized to the trans-Golgi network, but it is also detected in some other post Golgi compartments [21]. However, when cells are exposed to increased copper concentrations, ATP7A is trafficked to the plasma membrane and its main role is to prevent toxic intracellular accumulation of copper by expelling copper ions from the cell's interior [24], [25].

Mouse *Atp7a* mutants closely mimicking patients with Menkes disease serve as animal models of this inherited disorder and have recently been extensively used to explore metabolic copper-iron interactions [26], [27]. The *mosaic* mutation *(Atp7a^mo-ms^*) belongs to the group of *mottled* mutations, which severely affect copper metabolism, and is caused by mutation of the X-linked *Atp7a* gene. In *mosaic* mutants, a G to C nucleotide exchange in exon 15 of the *Atp7a* gene results in an arginine to proline substitution in the highly conserved 6^th^ transmembrane domain of the ATP7A protein. The mutated protein is mislocalized and is not translocated to the plasma membrane [28]. Hemizygous mutant males exhibit a severe phenotype including defects in pigmentation and hair structure, decreased body weight, and neurological problems such as ataxia, tremor and progressive paresis of the hind limbs. The *mosaic* mutation is usually lethal by day 17 of life [28].

Here, we analyzed potential changes in iron metabolism in copper-deficient 14-day old male *mosaic* mutant mice. The conception of our study was based on the assumption that copper deficiency may decrease the activity of Cu,Zn-superoxide dismutase (SOD1), an enzyme playing a crucial role in erythrocyte antioxidant defense, and thus may sensitize these cells to haemolysis, a pathological condition in which systemic iron metabolism is seriously affected [29]. Accordingly, we and others have previously demonstrated that mice with a disrupted *Sod1* gene are prone to haemolytic insult [30], [31]. Our results show that *mosaic* mutant mice exhibit some perturbations in systemic iron metabolism that are characteristic of moderate haemolysis.

## Materials and Methods

### Animals

Mice used in these experiments were bred in the Department of Genetics and Evolution, Jagiellonian University, and derived from a closed outbred colony. The animals were obtained by mating heterozygous *ms*/+ females with normal (+/−) males. The experimental material consisted of two groups of 14-day-old control (+/−) and mosaic mutant (*ms*/−) males. All mice were housed at constant temperature (22°C) under artificial light (12-hour photoperiod) and fed a standard Murigran diet (Motycz, Poland). Experiments were performed in accordance with Polish legal requirements under the licence of the First Local Ethical Committee on Animal Testing at the Jagiellonian University in Krakow (permission number: 85/2012). The animals were sacrificed by cervical dislocation.

### Blood and tissue collection

Blood samples were collected from a neck vein to EDTA-coated tubes. The EDTA-treated whole blood was used for the immediate preparation of smears. Blood samples were incubated at 4°C for 2 hours, and then serum and erythrocyte fractions were obtained by centrifugation at 2000×g for 10 min. The liver and kidneys were excised from mice following laparotomy. Tissue, serum and red blood cell (RBC) samples were immediately frozen in liquid nitrogen and stored at −80°C prior to molecular analysis.

### Measurement of erythrocyte copper content

The level of copper in erythrocytes from *ms/*− and *+/−* male mice was measured by atomic absorption spectrophotometry. Erythrocyte samples were weighed and digested in 2 ml of boiling Suprapur-grade nitric acid (Merck). After cooling to room temperature (RT), each sample was suspended in 10 ml of deionized water. Reference material samples were prepared in a similar manner. The copper concentration was measured using the graphite furnace AAS technique (AAnalyst 800, Perkin-Elmer). Three samples of nitric acid were used as blanks. In addition, three samples of a standard reference material, Cu = 189±4 mg/kg, were analyzed for normalization of the obtained data.

### Measurement of superoxide dismutase activity

SOD activity in erythrocyte total extracts was measured by gel electrophoresis using the Nitroblue Tetrazolium (NBT)/riboflavin method as described previously [32]. Briefly, 15 µg samples of the erythrocyte extracts were resolved by electrophoresis on 12% polyacrylamide gels under non-denaturing and non-reducing conditions. After electrophoresis, the activity of SOD was visualized by immersion of the gels in staining buffer [50 mM potassium phosphate (pH 7.8), 0.1 mM EDTA, 28 mM TEMED, 3 µM riboflavin, 0.25 mM NBT] for 30 min in the dark at RT. Gels were then exposed to light until the SOD activity bands became visible as bright bands on a dark blue background. The reaction was stopped by rinsing the gels with water and they were scanned and quantified immediately using a Molecular Imager with Quantity One software (Bio-Rad). The activities of Cu,Zn-SOD and Mn-SOD were distinguished by selective inhibition of the former activity by incubation of the gels in a buffer containing 3 mM KCN prior to staining, as described by Salin and Bridges [33].

### Immunoblot analysis

SOD1 was detected in total extracts (15 µg) obtained from peripheral erythrocytes using rabbit polyclonal anti-superoxide dismutase 1 antibody (Abcam; ab16831). To determine plasma haptoglobin (Hp) levels, 7 µl samples of 20-fold diluted mouse serum were used. Hp was detected with a chicken antibody raised against human Hp (US Biological). Its cross-reactivity with the mouse protein was demonstrated previously [31]. Ferritin levels in cytosolic extracts (20 µg) of mouse liver were analyzed using purified rabbit mouse liver ferritin antiserum (kindly provided by Dr. J. Brock, Glasgow University, Glasgow, UK) [34]. Haem oxygenase 1 (HO1) was detected in hepatic and renal crude membrane extracts (80 µg) using a rabbit polyclonal antibody raised against rat liver HO1 (StressGen). Actin, the loading control for serum and tissue extracts, was detected using an anti-human goat polyclonal antibody (Sigma; sc1615). Immunoreactive bands were disclosed using the ECL Plus Western blotting detection system (Amersham Life Sciences). Reactive bands were quantified relative to actin using a Molecular Imager with Quantity One software (Bio-Rad).

### Real-time Quantitative RT-PCR

HO1 and hepcidin (Hepc) hepatic mRNAs were measured by real-time quantitative RT-PCR as described previously [31]. Specific cDNA fragments were amplified using the following pairs of oligonucleotide primers: HO1, 5′-GTCGTGGTCAGTCAACATGG-3′ (forward) and 5′-TCTTGCCTGGCTCTCTTCTC-3′ (reverse); Hepc, 5′-CAATGTCTGCCCTGCTTTCT-3′ (forward) and 5′-TCTCCTGCTTCTCCTTG-3′ (reverse). The reactions were performed in a Light Cycler (Roche Diagnostics) and Light Cycler 3.5 Software was used for data analysis. Expression was quantified relative to that of a control transcript encoding glyceraldehyde-3-phosphate dehydrogenase [GAPDH; 5′-GACCACAGTCCATGCCATCAC-3′ (forward) and 5′-TCCACCACCCTGTTGCTGTAG-3′ (reverse)].

### Quantitative hepatic non-haem iron measurement

The non-haem iron content of liver fragments (100 mg) was determined by acid digestion of the samples at 100°C for 10 h, followed by colorimetric measurement of the absorbance of the iron-ferrozine complex at 560 nm as described previously [35].

### Immunofluorescence (IF) and confocal analysis of liver and kidney sections

After the sacrifice of mice, the liver and kidneys were immediately excised and fixed in 4% paraformaldehyde (Sigma) in phosphate-buffered saline (PBS) (Sigma) at 4°C for 24 h. After washing 3 times for 30 min in PBS, both tissues were successively soaked in 12.5 and 25% sucrose (Merck) for 1.5 and 12 h, respectively at 4°C. The tissues were then embedded in Tissue-Tek compound, frozen in liquid nitrogen and sectioned into 20-µm slices using a cryostat (Leica). The sections were washed in PBS and permeabilized by bathing in PBS/0.1% Triton X-100 (Sigma) for 10 min. Non-specific antibody binding was blocked by incubation of the tissue sections in PBS/3% BSA (Merck) for 1.5 h. For ferroportin (Fpn) detection in the liver, sections were incubated at RT with primary rabbit polyclonal anti-Fpn antibody (Alpha Diagnostic) diluted 1∶250 in PBS/3% BSA. The sections were then washed 3 times with PBS and incubated with Cy3 (indocarbocyanine)-conjugated goat anti-rabbit antibody (Jackson Immunoresearch) diluted 1∶500 in PBS/3% BSA. For haem oxygenase 1 (HO1) detection, liver and kidney sections were prepared as described above and incubated overnight at RT with primary rabbit polyclonal anti-HO1 antibody (StressGen) diluted 1∶250 in PBS/3% BSA. The sections were washed 3 times with PBS and then incubated with Cy3-conjugated goat anti-rabbit antibody diluted 1∶500 in PBS/3% BSA. In the liver we also performed co-labeling of HO1 and Fpn with F4/80, a membrane macrophage marker [36]. Liver sections were incubated overnight with either anti-Fpn or with anti-HO-1 antibody and rat anti-mouse F4/80 monoclonal antibody (Serotec) diluted 1∶500 in PBS/3% BSA. The sections were then incubated with a mixture of Cy3-conjugated goat anti-rabbit antibody and Alexa 488 goat anti-rat antibody (Jackson Immunoresearch) in PBS/3% BSA for 1 h. Finally, the sections were washed 3 times for 10 min in PBS at RT and mounted using Vectashield with 4′,6-diamidine-2-phenylindole (DAPI; Vector Labs). As a negative control, some sections were prepared without incubating with primary antibody. IF was analyzed with a Zeiss LSM 510 Meta confocal microscope (Carl Zeiss, Jena, Germany) using the 60x objective.

### Statistical Analysis

Statistical analysis was performed using a two-tailed Student's *t* test, with *p* values of <0.05 and <0.01 being considered statistically significant and highly significant, respectively.

## Results

### Copper deficiency and decreased SOD1 activity and expression in erythrocytes of mutant *ms/−* mice

**Figure 1:**
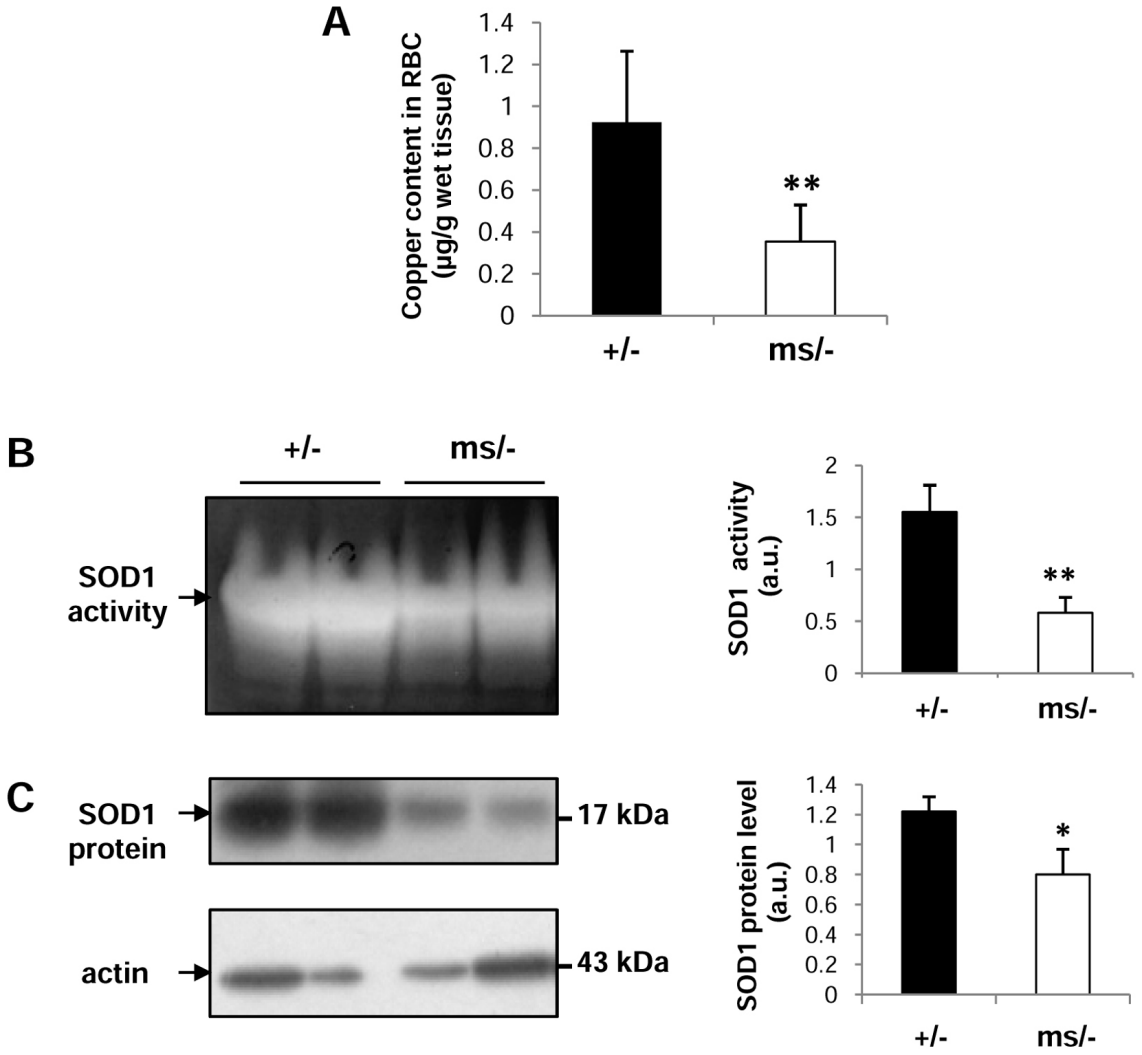
Decreased copper content and activity/expression of SOD1 in circulating erythrocytes from *ms/−* mice. (**A**) Decreased copper content in erythrocytes of *ms/−* mice. Values are expressed as the means ± S.D. for erythrocyte samples obtained from 15 control (*+/−*) and 15 mutant (*ms/−*) males. (**B**) *left-hand panel*, the activity of SOD was measured after resolution by gel electrophoresis using the Nitroblue Tetrazolium (NBT)/riboflavin method as described in the Experimental section. The analyses were performed using erythrocyte total extracts obtained from *ms/−* and *+/−* males and representative results are shown. (**C**) *left-hand panel*, SOD1 levels in erythrocytes were analyzed by western blotting as described in the Experimental section. The analyses were performed using erythrocyte total extracts obtained from *ms/−* and control (*+/−*) males, and representative results are shown. The blot was reprobed with monoclonal anti-actin antibody as a loading control. (**B,C**) *right-hand panels*, the intensity of the SOD bands was quantified with a molecular Imager using Quantity One software (Bio-Rad) and is plotted in arbitrary units to present activity (**B**) and protein level (**C**). Results are expressed as the mean ± S.D. for 5 mice of both the *ms/−* and *+/−* genotypes. Significant differences are indicated (* – P<0.05; ** – P<0.01).

14-day-old *mosaic* mutant mouse males exhibit a tissue copper concentration pattern typical of Menkes disease, i.e. reduced levels in both liver and brain and increased levels in both kidney and duodenum [37], [38]. Here, we analyzed for the first time the copper content in circulating RBCs of such mutants and found that it was nearly 60% lower compared to wild-type animals (Figure 1A). Bearing in mind that the bioavailability of copper is crucial for maintaining SOD1 activity at the required level [39], we then assessed the activity of this key antioxidant enzyme in erythrocytes of both mutant and wild-type males. Similarly to the copper concentration, SOD1 activity in erythrocytes measured by the NBT/riboflavin method [32] was substantially reduced in *ms/−* mice as shown by densitometry of SOD1 bands (Figure 1B). Moreover, we found that this decrease was at least partly due to the reduced SOD1 protein level in *mosaic* erythrocytes, detected by western immunoblotting (Figure 1C).

### Haemolysis and haemoglobin (Hb)-scavenging systems in mutant *ms/−* mice

**Figure 2:**
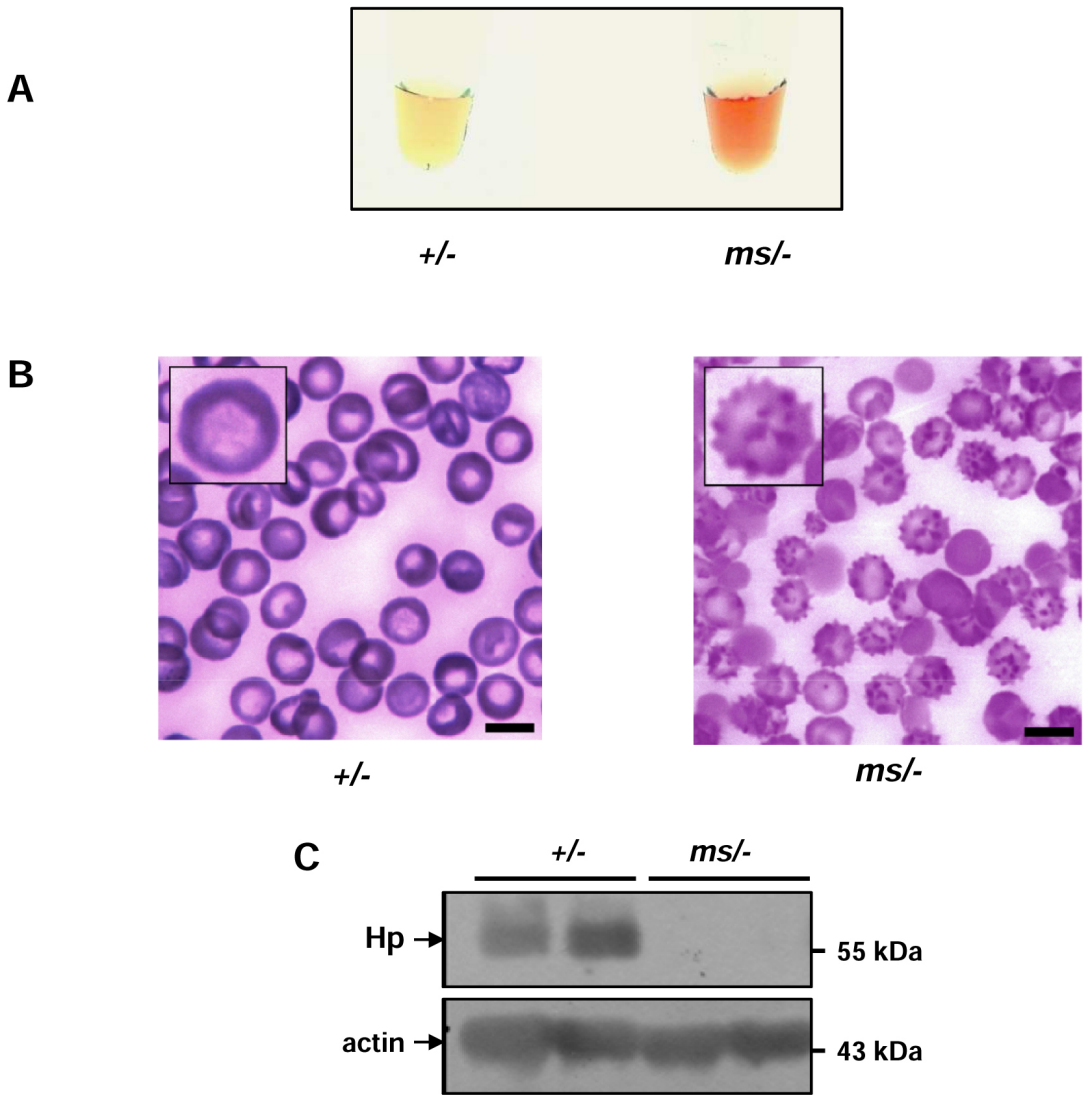
Haemolysis and acanthocytosis in *ms/−* mice. (**A**) Serum of control (*+/−*) and mutant (*ms/−*) males obtained by centrifugation. (**B**) Peripheral blood smears of *ms/−* mice showing increased numbers of acanthocytes. High magnification images of a normal and an abnormally-shaped erythrocyte are shown (insets). Six animals from both the control and *ms/−* male groups were examined and one typical sample of each is shown. Bars correspond to 5 µm. (**C**) Plasma levels of haptoglobin (Hp) were assessed by western blotting as described in the Experimental section. The blot was reprobed with monoclonal anti-actin antibody as a loading control.

We and others have shown that in SOD1 knock-out (KO) mice, erythrocytes become sensitive to rupture due to oxidative stress and, in consequence, KO SOD1 mice display clear signs of haemolysis [30], [31]. Therefore, in the present study we attempted to identify hallmarks of intravascular haemolysis in *ms/−* mice. It was immediately noticeable that serum obtained from the blood of *ms/−* mice, centrifuged immediately after neck vein puncture, was a reddish color (Figure 2A). Wright staining of blood smears from *ms/−* mice revealed a large percentage of irregularly-shaped RBCs, the morphology of which strongly resembled that of acanthocytes (Figure 2B). We then examined the serum level of haptoglobin (Hp), an acute phase protein that traps free Hb released from ruptured erythrocytes and eliminates it from the circulation [40], [41]. In contrast to free Hp, the stable Hp-Hb complex binds the CD163 receptor with high affinity and is rapidly delivered mainly to the reticulo-endothelial system by endocytosis [40], [41]. Therefore, a decrease in the Hp concentration in the serum is generally considered a marker of accelerated haemolysis [40], [41]. Western blot analysis showed that Hp was completely depleted in the plasma of *ms/−* mice (Figure 2C), suggesting intensive plasma clearance of free Hb.

### Increase in hepatic and renal haem oxygenase 1 (HO1) expression in mutant *ms/−* mice

**Figure 3:**
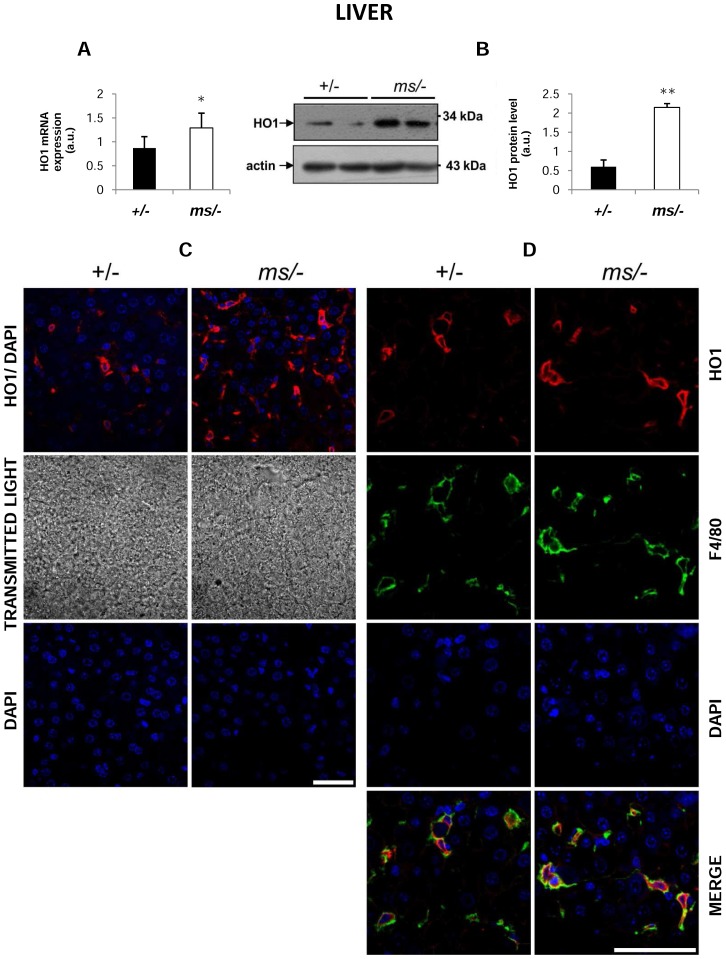
Increased expression of haem oxygenase 1 (HO1) in Kupffer cells of *ms/−* mice. (**A**) Real-time quantitative PCR analysis of hepatic HO1 mRNA expression. The histogram displays HO1 mRNA levels in arbitrary units (means ± S.D., n = 6). (**B**) *left-hand panel* Western blot analysis of HO1 protein levels in hepatic membrane fractions prepared from *+/−* and *ms/−* males. The blot was reprobed with polyclonal anti-human actin antibody as a loading control. *right-hand panel* Immunolabelled HO1 bands from six mice were quantified using a Molecular Imager and HO1 protein levels (means ± S.D.) are plotted in arbitrary units. * – P<0.05. (**C**) *top panel* Immunofluorescent staining of HO1 in *+/−* and *ms/−* livers analyzed by confocal microscopy. *middle panel* Tissue morphology observed in transmitted light. *bottom panel* To confirm the specificity of Fpn detection, liver sections of *+/−* and *ms/−* males were incubated with only the secondary antibody. No HO1 staining was detected in these negative controls. Nuclei were counterstained with DAPI. Bars correspond to 50 µm. (**D**) Colocalization (*bottom panel*) of HO1 (red channel) and F4/80, a macrophage marker (green channel) in the livers of *+/*− and *ms/−* males analyzed by confocal microscopy. Nuclei were counterstained with DAPI. Bars correspond to 50 µm.

**Figure 4:**
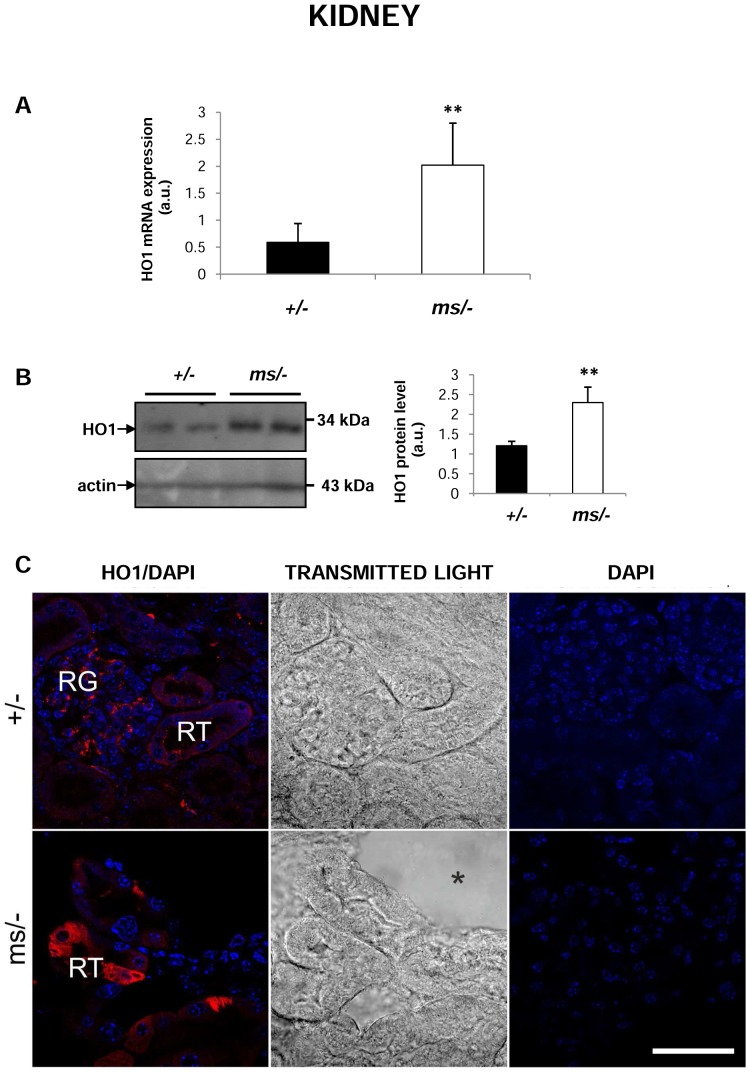
Increased expression of haem oxygenase 1 (HO1) in kidneys of *ms/−* mice. (**A**) Real-time quantitative PCR analysis of renal HO1 mRNA expression. The histogram displays HO1 mRNA levels in arbitrary units (means ± S.D., n = 6). (**B**) *left-hand panel* Western blot analysis of HO1 protein levels in hepatic membrane fractions prepared from *+/−* and *ms/−* males. The blot was reprobed with polyclonal anti-human actin antibody as a loading control. *right-hand panel* Immunolabelled HO1 bands from six mice were quantified using a Molecular Imager and HO1 protein levels (means ± S.D.) are plotted in arbitrary units. ** – P<0.01. (**C**) *left-hand panel* Immunofluorescent staining of HO1 in *+/−* and *ms/−* kidneys analyzed by confocal microscopy. RN – renal tubules; RG – renal glomeruli. *middle panel* Transmitted light image shows the structure of glomeruli and tubules as well as the presence of a large lesion (asterisk) in the kidney of a mutant. *right-hand panel* To confirm the specificity of HO1 detection, kidney sections of *+/−* and *ms/−* males were incubated with only the secondary antibody. No HO1 staining was detected in these negative controls. Nuclei were counterstained with DAPI. Bars correspond to 50 µm.

Hb produced by haemolysis is recovered from the circulation by the liver in a process that entails the transcriptional induction of HO1 expression [42], which represents the most important protective system against haem toxicity. A comprehensive assessment of HO1 expression and localization in the liver clearly showed significant induction of the *Hmox1* gene (coding HO1) at both the mRNA (Figure 3A) and protein levels (Figure 3B, 3C and 3D) in *mosaic* mutants. Co-localization studies demonstrated that HO1 was mainly located in Kupffer cells in both control and mutant males (identified by staining with the anti-F4/80 antibody specific for a macrophage membrane marker [36]) (Figure 3D). Quantitative RT-PCR (Figure 4A) and western blotting (Figure 4B) analyses showed that HO1 expression was also increased in the kidney of *ms/−* mice. Microscopic analysis of immunofluorescent (IF) staining and transmitted light images indicated that expression of HO1 in the renal cortex of control males expression of HO1 occurred mainly in the renal glomeruli (Figure 4 C, upper panel), whereas in the mutant males, HO1 expression was strongly up-regulated in the cells of renal tubules (Figure 4C, bottom panel).

### Decreased ferroportin (Fpn) and increased hepcidin (Hepc) expression in the liver of *ms/−* mice

**Figure 5:**
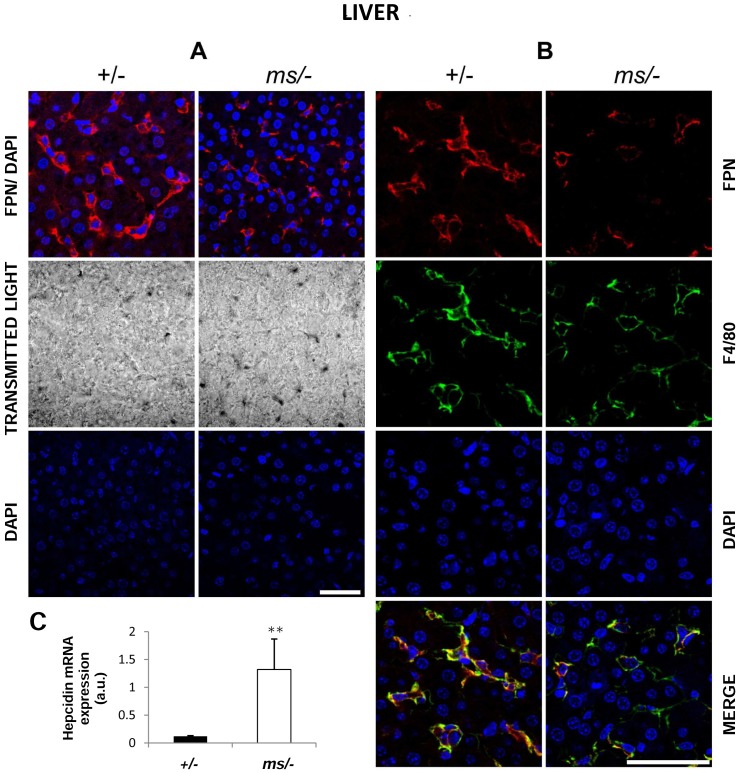
Correlation between decreased ferroportin (Fpn) protein level and increased hepcidin (Hepc) mRNA expression in the liver of *ms/−* males. (**A**) Immunofluorescent staining of Fpn in *+/−* and *ms/−* males. *top panel* Livers analyzed by confocal microscopy. *middle panel* Tissue morphology observed in transmitted light. *bottom panel* To confirm the specificity of Fpn detection, liver sections of *+/−* and *ms/−* males were incubated with only the secondary antibody. No Fpn staining was detected in these negative controls. Nuclei were counterstained with DAPI. Bars correspond to 50 µm. (**B**) Colocalization (*bottom panel*) of Fpn (red channel) and F4/80, a macrophage marker (green channel) in liver from *+/−* and *ms/−* males analyzed by confocal microscopy. Nuclei were counterstained with DAPI. Bars correspond to 50 µm. (**C**) Real-time quantitative PCR analysis of hepatic Hepc mRNA expression in *+/−* and *ms/−* males. The histogram displays Hepc mRNA levels in arbitrary units (means ± S.D., n = 6). Significant difference is indicated (** – P<0.01).

Assessment of the functioning of the Hepc-Fpn axis is an indispensable element in the analysis of iron metabolism. It has been reported that Fpn, the sole cellular iron exporter known in mammalian cells [7], is up-regulated in the liver in various mouse models of haemolysis [29], [31]. Using IF detection we found substantial hepatic Fpn down-regulation in *ms/−* males compared with control animals (Figure 5A). Confocal analysis of dual staining with antibodies specific for Fpn and the plasma membrane macrophage marker F4/80, indicated strong co-localization, suggesting that Fpn is located in liver macrophages (Kupffer cells) in both wild-type and *mosaic* mutant males (Figure 5B). Detailed analysis of the microscopic images revealed strong cell membrane localization of Fpn in control animals (Fpn staining precisely overlaps that of F4/80), whereas membrane expression of Fpn on Kupffer cells appeared much weaker in the mutants (Figure 5B, bottom panel). Quantitative RT-PCR analysis showed an increase in the Hepc mRNA level of about 10-fold in the livers of *ms/−* mice compared with those of *+/−* animals. Assuming that systemic inflammation may be responsible for the transcriptional induction of *Hamp* gene expression, it is noteworthy that the kidneys of *ms/−* males showed clear signs of inflammation including cellular vacuolization, necrosis of the renal tubule epithelial cells, lymphocyte infiltration, and damage to renal glomeruli structures [43], [44].

### Hepatic and renal iron status in *ms/−* mice

**Figure 6:**
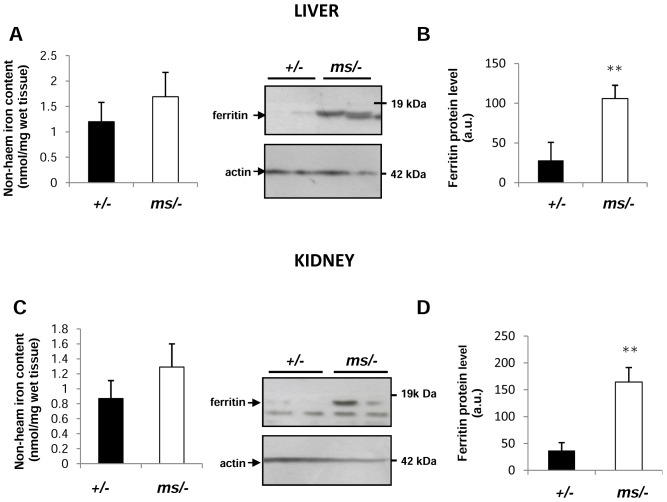
Hepatic and renal iron status in control (+/−) and mutant (*ms/−*) mice. Non-haem hepatic (**A**) and renal (**C**) iron content was measured as described in the Experimental section. Values are expressed as the means ± S.D. for both liver and kidney samples obtained from 15 mice of each genotype. L-ferritin levels in hepatic (**B**) and renal (**D**) cytosolic protein extracts (50 µg/lane) were assessed by western blot analysis *left-hand panels*. The blots were reprobed with polyclonal anti-human actin antibody as a loading control. (**B**) and (**D**) *right-hand panels* Immunolabelled ferritin bands from four mice were quantified using a Molecular Imager and ferritin protein levels (means ± S.D.) are plotted in arbitrary units. ** – P<0.01.

In the context of accelerated Hb clearance and the enhanced capacity to degrade excess haem in the liver and kidneys of *ms/−* males, we investigated the consequences of these activities on the iron status in both organs. Although our data showed a tendency for hepatic and renal iron accumulation in *ms/−* males compared with *+/−* animals (Figure 6A and 6C), the differences in non-haem iron content between these mice were not significant (P values >0.05). In general, the ferritin level serves as a marker of iron loading in cells and tissues [45]. Moreover, it is usually elevated in the kidneys and liver under conditions of haemolysis [29], [46]. Analysis of liver and kidney cytosolic extracts by western blotting revealed significantly higher ferritin expression in *ms/−* males compared with the control animals (Figure 6B and 6D).

## Discussion

The interaction between the multi-copper ferroxidases caeruloplasmin and hepheastin, and ferroportin in the egress of iron from cells is a major biological link between copper and iron, and the best characterized at the molecular level [3]. It is assumed that mutation of genes encoding caeruloplasmin and hephaestin as well as copper deficiency, which decreases the ferroxidase activity of both enzymes, reduces the amount of iron available for erythropoiesis and contributes to the accumulation of iron in the liver, intestine and brain [3]. Another important interaction between these two biometals is copper-dependent iron utilization for haemoglobin synthesis by erythroid cells [18], but the molecular nature of this process is poorly understood [47]. Recently, an additional copper-iron relationship was identified in mice under conditions of nutritional copper deficiency [48]. This involves a series of consecutive events such as iron-deficiency anaemia, hypoxia, stabilization of HIF2-α in duodenal enterocytes and subsequent transcriptional up-regulation of genes responsible for enhanced iron absorption [48].

In the present study, we examined the adaptive response of systemic iron regulation to copper deficiency, which causes altered activity of the first-line defence antioxidant cuproenzyme, superoxide dismutase 1 (SOD1, Cu,Zn-SOD), a scavenger of the superoxide anion radical (O_2_ **^.^** ^−^) [49]. Previous study performed on 10 and 11-day-old mice showed that both dietary copper deficiency in wild-type mice and copper insufficiency in *brindled* mutant mice (*Atp7a^mo-br^*) lead to the decrease in SOD1 activity in many organs such as liver, spleen, brain, heart, thymus and bone marrow [50]. Decrease in SOD1 activity was also described in organs of 13-day old *brindled* and *blotchy* (*Atp7a^mo-blo^*) mutant males [51]. This study was designed to test whether copper deficiency is responsible for decreased SOD1 activity in erythrocytes, resulting in membrane damage, haemolysis and subsequent changes in systemic iron metabolism. This hypothesis was formulated in the light of the following information: 1) SOD1-associated copper accounts for more than half of the total copper content in circulating erythrocytes [52]; 2) nutritional copper deficiency has been shown to decrease both the expression and activity of SOD1 in erythrocytes [39]; 3) we [31] and others [30] have demonstrated the occurrence of haemolysis in mice lacking Cu,Zn-SOD; and 4) haemolysis entails substantial changes in iron metabolism [29].

In our experiments we used a well characterized *mosaic* mouse line, representing a model of Menkes disease, which carries a mutation in the *Atp7a* gene and therefore lacks the ATP7A copper-transporting P-type ATPase [28]. When untreated with copper, *mosaic* mutant hemizygous males die by day 17 of life [28] and so we used 14-day-old animals in our study. Their pattern of body copper content and distribution is typical for Menkes disease, i.e. increased copper accumulation in the small intestine and kidneys, and severe copper deficiency in the brain, liver and heart [37], [38]. To our knowledge, erythrocyte copper content has never been described in patients with Menkes disease or in mouse models of this condition. Considering that the duodenal ATP7A transporter plays a crucial role in copper uptake to the organism [21], it is plausible that its dysfunction in *ms/−* male mice may result in impaired copper delivery to erythroid precursors and in consequence, low copper levels in peripheral erythrocytes. Indeed, measurement of the copper concentration in circulating *ms/−* erythrocytes showed a decrease of about 60% compared with that of healthy controls. This decrease correlated strongly with the down-regulation of both SOD1 expression (30%) and activity (70%). A reduction in erythrocyte SOD1 activity in response to dietary copper deficiency has been described in various mammalian species [53]–[55] including mice [39]. Obviously, altered functioning of Cu,Zn-SOD increases the risk of oxidative stress. With regard to erythrocytes, this phenomenon is well documented in SOD1 null mice, in which the lack of Cu,Zn-SOD activity is responsible for increased oxidative stress in circulating erythrocytes [30], [56] and the production of autoantibodies against erythrocytes [30]. These events were found to be causally connected with the increased sensitivity of erythrocytes to oxidative injury and haemolysis [30], [31]. Although *ms/−* erythrocytes still display about 30% of normal SOD1 activity, the serum obtained from these animals clearly has a haemolytic appearance. The threshold level of SOD1 activity required to protect erythrocytes against oxidative stress is unknown, although it was previously shown that a decrease in SOD1 activity in *Sod1^+/−^* erythrocytes to half of the value in *Sod1^+/+^* cells is still sufficient to ensure the protective activity of the enzyme [56]. Interestingly, *ms/−* male mice have a large number of acanthocytes (about 40%) in the peripheral blood. Acanthocytosis may occur due to alteration of the lipid composition and fluidity of the red cell membrane, but can also be caused by dehydration in a subpopulation of acanthocytic erythrocytes. Indeed, *mosaic* suckling males show macroscopic signs of dehydration such as low body weight, loss of liquids due to diarrhea, and increased density of the plasma. It is worth noting that mild to moderate haemolysis was reported to be associated with acanthocytosis [57]. One of the valuable hallmarks of intravascular haemolysis is the disappearance of haptoglobin (Hp) from the serum [40], [41]. Hp is an acute-phase protein involved in the clearance of haemoglobin (Hb) from the bloodstream to prevent its toxicity [40], [41]. When Hb is released from ruptured erythrocytes, it is instantly bound by Hp and forms a stable Hp-Hb complex [40], [41], which is then rapidly removed from the circulation by binding to the CD163 receptor, present mainly on tissue macrophages including Kupffer cells [58]. Since free Hp does not bind to CD163, specific recognition of the Hp-Hb complex by this receptor explains the decrease in Hp concentration in the plasma during accelerated haemolysis [40], [41]. However, in contrast to humans, the mouse CD163 may also directly bind Hb that is not complexed with Hp [59]. Nevertheless, we found that the serum of *ms/−* mice was completely depleted of Hp, suggesting that the Hb scavenging mechanism functions very efficiently in these mutant males. The next steps of Hb inactivation are its lysosomal degradation followed by haem breakdown catalyzed by haem oxygenase 1 (HO1) [reviewed in 42]. Besides functioning as a substrate of HO1, haem is known to be the most potent transcriptional inducer of the *Hmox1* gene [60]. Unsurprisingly, increases in the level of both the HO1 transcript and protein were detected in the liver of *ms/−* mutants. Significantly, we previously found that the expression of HO1 was also strongly induced in the liver of *Sod1^−/−^* mice displaying moderate haemolytic anaemia [31]. Furthermore, transcriptional induction of hepatic HO1 was observed under conditions of intravascular haemolysis in β-thalassemic and sickle mice [29] as well as during malaria [61]. The identification of Kupffer cells as a site of hepatic HO1 expression in *ms/−* mice strongly suggests that these cells are mostly charged with the detoxification of haem derived from Hb taken up from the bloodstream. HO1 also appears to be largely responsible for haem catabolism during erythrophagocytosis [62]. Therefore, it is noteworthy that in agreement with the subcellular localization of HO1 found in mouse bone marrow-derived macrophages during erythrophagocytosis [62], immunostaining of this enzyme was predominantly intracytosolic in the Kupffer cells of *ms/−* mice.

Studies on Hp-null mice showed that even under conditions of physiological haemolysis, Hb is filtered by the kidneys [63]. Renal filtration of Hb also occurs in normal subjects (mice and humans) when the buffering capacity of Hp is overwhelmed in acute haemolysis [40], [41]. Studies in disease models demonstrated that the kidney can adapt to increased amounts of Hb by inducing HO1 [63]. Such a situation seems to occur in *ms/−* mice. Indeed, in the kidneys of these animals we showed a marked induction of HO1 in cells of the renal tubules, very possibly as a consequence of glomerular filtration of Hb. However, it is also possible that the *Hmox1* gene is induced in *ms/−* mouse kidney in response to an inflammatory reaction, the occurrence of which has been well documented in the kidneys of 14-day-old *mosaic* males [43], [44]. It has recently been claimed that the protective antioxidant effect of HO1 depends, at least in part, on co-induction of ferritin, a cytosolic protein that binds, stores and thus detoxifies reactive iron released by haem breakdown [64]. Accordingly, in both the liver and kidneys of *ms/−* males, ferritin was elevated at the protein level compared to their healthy littermates. However, the *Hmox1* gene might also be induced in the kidneys of *ms/−* mice in response to the inflammatory reaction caused by excessive copper accumulation. Available data suggest a likely mechanism for this copper overload. In the kidneys of healthy animals, copper reabsorption from the urine occurs via the proximal renal tubules [65], [66]. Most of copper ions are then transferred back to the circulation *via* ATP7A located in the basolateral membranes of epithelial cells of these tubules [67]. This explains why dysfunction of ATP7A protein in *Atp7a* mutants results in toxic copper accumulation at the renal-proximal epithelium, a phenomenon detected in various mouse models of Menkes disease [65], [66], including *mosaic* mice [43], [44].

Apart from the induction of the Hp-CD163-HO1 pathway [42], the adaptive changes in the iron metabolism under haemolytic conditions also involve the redistribution of iron from macrophages back to the circulation. This process is mediated by ferroportin (Fpn), the sole cellular exporter of ionic iron known in mammalian cells [7]. We previously demonstrated that enhanced expression of hepatic Fpn in haemolytic anemia in KO SOD1 mice ensures the iron supply required for erythropoiesis [31]. Surprisingly, in *ms/−* mice, Fpn was down-regulated in Kupffer cells, the principal cell type expressing Fpn in the liver. According to the consensus of the hepcidin-ferroportin regulatory axis [7], such down-regulation of Fpn expression strongly suggested the concomitant up-regulation of hepatic hepcidin. Indeed, hepcidin was elevated at the mRNA level by more than 10-fold in *ms/−* mutants compared with control males. We hypothesize that the renal inflammation induced in *mosaic* mice by heavy copper overload [38], [43], [44] diffuses throughout the organism of *ms/−* mice and mediates transcriptional activation of the *Hamp* gene in the liver. Accordingly, it is well established that plasma hepcidin levels remain persistently high in patients with chronic kidney disease [68]. Furthermore, inflammatory mediators such as interleukin-6 and -1 participate in the iron-independent induction of the *Hamp* gene [69]. Despite increased and decreased expression of HO1 and Fpn, respectively, we observed only a tendency in both liver and kidney of *ms/−* mice to accumulate non-haem iron, although the elevated level of ferritin in both organs seems to support some retention of iron in these tissues. It is possible that the extent of iron loading in the two examined organs is quite limited. A similar lack of global hepatic iron loading has also been reported in haemolytic mice lacking Cu,Zn-SOD1 [31].

In summary, we have identified a new indirect relationship between copper deficiency in the mouse model of Menkes disease and systemic iron metabolism. The key point in this interconnection is decreased activity of the erythrocyte antioxidant cuproenzyme Cu,Zn-SOD, resulting in oxidative stress, increased permeability of erythrocyte membranes and haemolysis. Hb released from damaged erythrocytes causes the mobilization of a protective system neutralizing Hb toxicity, including Hp-dependent elimination of Hb from the circulation as well as the degradation of haem derived from Hb by HO1 in the liver and kidneys. Importantly, our results show for the first time that suckling mice adapt their iron metabolism to haemolytic insult in a similar manner to adult animals. Finally, haemolysis has been identified as an additional pathological symptom in a mouse model of Menkes diseases.

It is largely known that similarly to *mosaic* mutants [44], Menkes patients suffer from kidney and urinary tract dysfunctions and develop chronic renal inflammation [70]. Increased hepcidin synthesis under these pathological conditions may reduce availability of iron for erythropoiesis and in combination with haemolysis may contribute for the development of anemia. Taking together, our results point out a need to monitor iron status in Menkes patients.
